# *Siegesbeckia orientalis* Extract Inhibits TGFβ1-Induced Migration and Invasion of Endometrial Cancer Cells

**DOI:** 10.3390/molecules21081021

**Published:** 2016-08-05

**Authors:** Chi-Chang Chang, Xue-Hua Ling, Hsia-Fen Hsu, Jing-Mei Wu, Chao-Ping Wang, Jyh-Ferng Yang, Li-Wen Fang, Jer-Yiing Houng

**Affiliations:** 1 Department of Obstetrics & Gynecology, E-Da Hospital/I-Shou University, Kaohsiung 82445, Taiwan; ed101779@edah.org.tw (C.-C.C.); ed107312@gmail.com (X.-H.L.); jingmei0112@hotmail.com (J.-M.W.); 2 Graduate Institute of Biotechnology and Chemical Engineering, I-Shou University, Kaohsiung 84001, Taiwan; jfyang@isu.edu.tw; 3 Department of Nutrition, I-Shou University, Kaohsiung 82445, Taiwan; fen153848@gmail.com (H.-F.H.); fanglw@isu.edu.tw (L.-W.F.); 4 Division of Cardiology, Department of Internal Medicine, E-Da Hospital/I-Shou University, Kaohsiung 82445, Taiwan; ed100232@edah.org.tw

**Keywords:** *Siegesbeckia orientalis* linne, endometrial cancer, anti-metastasis

## Abstract

Type II endometrial carcinoma typically exhibits aggressive metastasis and results in a poor prognosis. *Siegesbeckia orientalis* Linne is a traditional Chinese medicinal herb with several medicinal benefits, including the cytotoxicity against various cancers. This study investigates the inhibitory effects of *S. orientalis* ethanol extract (SOE) on the migration and invasion of endometrial cancer cells, which were stimulated by transforming growth factor β (TGFβ). The inhibitory effects were evaluated by determining wound healing and performing the Boyden chamber assay. This study reveals that SOE can inhibit TGFβ1-induced cell wound healing, cell migration, and cell invasion in a dose-dependent manner in RL95-2 and HEC-1A endometrial cancer cells. SOE also reversed the TGFβ1-induced epithelial-mesenchymal transition, including the loss of the cell-cell junction and the lamellipodia-like structures. Western blot analysis revealed that SOE inhibited the phosphorylation of ERK1/2, JNK1/2, and Akt, as well as the expression of MMP-9, MMP-2, and u-PA in RL95-2 cells dose-dependently. The results of this investigation suggest that SOE is a potential anti-metastatic agent against human endometrial tumors.

## 1. Introduction

Endometrial cancer is a common gynecologic malignancy in the Western world [1,2]. In Taiwan, the incidence of endometrial cancer is second only to that of cervical cancer in women and the number of cases is rising gradually [3]. Endometrial cancer can be classified into Type I and Type II based on clinical behavior and morphological phenotype. Type I cases are low-stage and low-grade, while Type II cases are advanced-stage and aggressive [4]. Most cases of Type I can be diagnosed at an early stage because some symptoms would appear in this stage, and such cases present a high survival percentage following primary surgery. In contrast, Type II patients typically have a poor prognosis because the carcinoma has an aggressive phenotype that is characterized by lymphovascular invasion, high histological grade, and myometrial invasion, resulting in distant metastases via the hematogenous route [5].

Transforming growth factor β (TGFβ) signaling has been identified as crucial in the initial steps of endometrial carcinoma invasion and metastasis [6]. The function of TGFβ in tumor biology is complex. It suppresses tumor activity in the early stages of carcinogenesis, but becomes a tumor promoter in the later stages [7]. Three TGFβ isoforms (β1, β2, and β3) exists in human endometrial tumors. TGFβ1 can activate tumor promotion and the epithelial-to-mesenchymal transition (EMT), which makes tumor cells move away from their epithelial cell community and integrate into surrounding tissue [8,9,10]. Clinical studies have shown that a significant increase in levels of TGFβ1 in the serum of patients with breast cancer, lung cancer, hepatoma, prostate cancer, and stage I and stage II endometrial carcinoma [11,12]. The overexpression of TGFβ1 in endometrial cancer cells correlates with tumor metastasis and a poor patient outcome [13,14].

For patients in an advanced or later stage endometrial cancer, despite surgery, treatment includes chemotherapy, radiotherapy, and hormonal treatment [15]. Chemotherapy with cisplatin, doxorubicin, and paclitaxel has been found to be superior to radiotherapy [16]. However, the effectiveness of these strategies remains limited. Recently, several reports have suggested that Chinese herbal medicines are effective in delaying tumor progression, preventing recurrence and metastasis, alleviating clinical symptoms, improving immune function, increasing the quality of life, and prolonging the life span of cancer patients [17,18].

*Siegesbeckia orientalis* L. is a member of the Asteraceae family. As a potential and popular folk-medicine herb, *S. orientalis* has been prescribed for treating snakebites, cutaneous disorders, rheumatic arthritis [19,20], allergies [21], and immune deficiency [22], and it is also taken orally as an anti-inflammation and anti-cancer agent [23,24]. The authors’ laboratory has confirmed, using in vitro and in vivo models, that the ethanol extract of *S. orientalis* (SOE) can attenuate acute inflammation by inhibiting inflammatory mediators through the suppression of MAPKs- and NF-κB-dependent pathways [25]. We have also demonstrated that SOE inhibits the growth of RL95-2 human endometrial cancer cells in a dose-dependent manner, and this effect is associated with G2/M phase cell cycle arrest, activation of caspase-3, -8, and -9, upregulation of Bad, Bak, and Bax, and downregulation of Bcl-2 and Bcl-xL [26]. The present study investigates the inhibitory effects of SOE on the motility and invasion of endometrial cancer cells under the stimulation by TGFβ1. The effects of SOE on the expression of MMPs, and the activities of MAPK and Akt were also examined.

## 2. Results and Discussion

### 2.1. Effect of SOE on Morphology of Endometrial Cancer Cells

**Figure 1:**
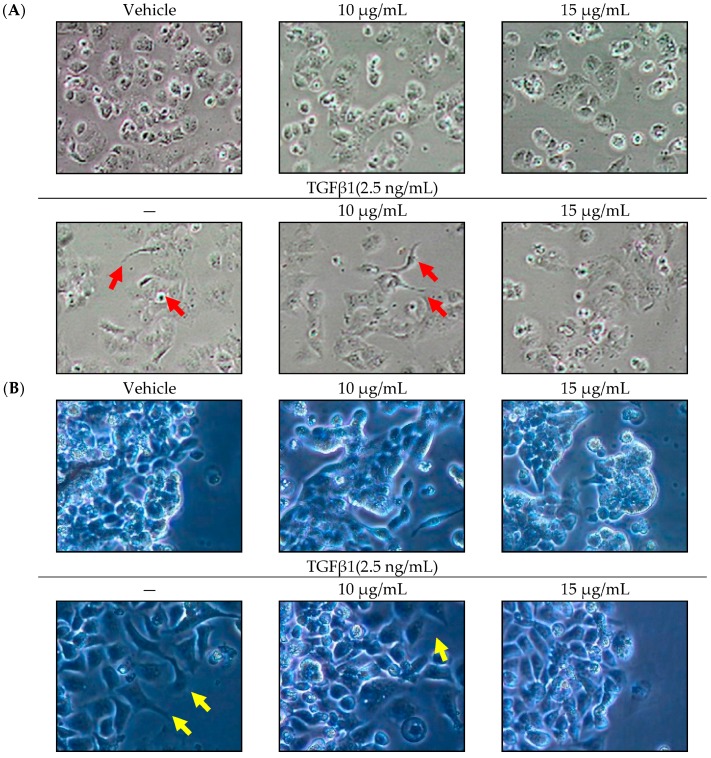
Changes of morphology of endometrial cancer cells in absence or presence of SOE for 24 h of incubation. (**A**) HEC-1A cells and (**B**) RL95-2 cells. Photomicrographs of culture plates were directly obtained under a phase-contrast microscope (Nikon Eclipse TS100, Chiyoda-ku, Tokyo, Japan; magnification 20×). Arrow indicates lamellipodia-like structure.

TGFβ1 may result in the acquisition of an invasive phenotype of endometrial carcinoma. The mesenchymal phenotype is an important characteristic of EMT and is associated with invasive and metastatic effects [6]. As shown in Figure 1, when 2.5 ng/mL of TGFβ1 was added to endometrial cancer cells, the mesenchymal phenotype was changed, including the loss of the cell-cell junction and the formation of a lamellipodia-like structure. Under the induction by TGFβ1, the treatment of SOE reversed the mesenchymal phenotypes of HEC-1A and RL95-2 cancer cells.

### 2.2. Inhibitory Effect of SOE on Proliferation of Endometrial Cancer Cells

**Figure 2:**
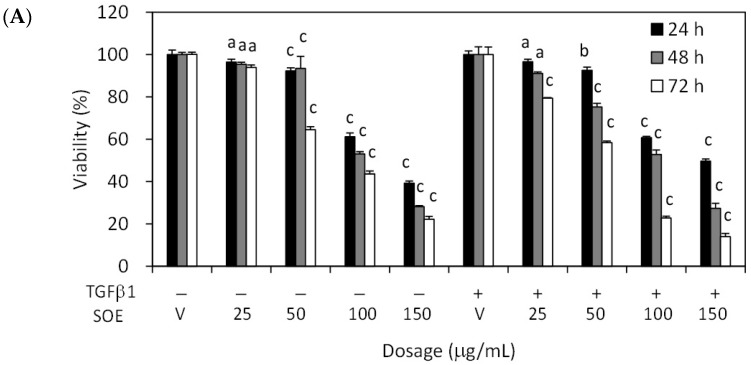

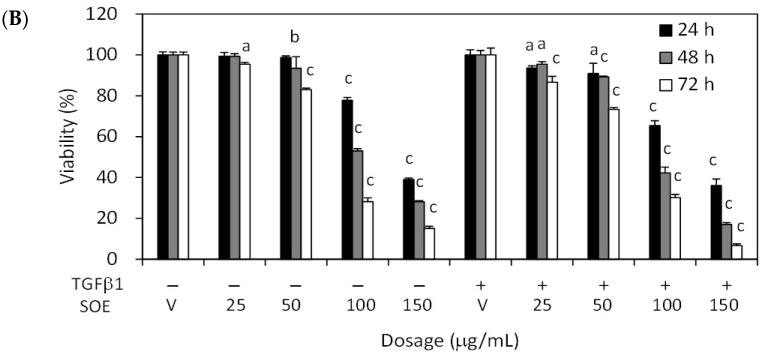
Effects of concentration and duration of SOE treatment on proliferation of endometrial cancer cells, with or without TGFβ1 (2.5 ng/mL) stimulation. (**A**) HEC-1A cells and (**B**) RL95-2 cells. Cancer cells were cultured in 96-well plates at a density of 1 × 10^4^ cells/well. Cells were treated with 0–150 μg/mL SOE for 24–72 h. Cell viability was measured using 3-(4,5-dimethylthiazol-2-yl)-2,5-diphenyltetrazolium bromide (MTT) assay. Three independent experiments were conducted; results are expressed as mean ± SD. A significant difference from the respective vehicle, according to Student’s *t-*test, was indicated as “a” for *p* < 0.05; “b” for *p* < 0.01, and “c” for *p* < 0.001.

The inhibitory effects of treatment with various SOE concentrations for various incubation durations on the viability of HEC-1A and RL95-2 cells were examined. Figure 2 reveals that SOE, with or without TGFβ1 induction, inhibited the viability of both cell lines in dose- and time-dependent manner. Treatment for 24–48 h with SOE concentrations of 0 to 25 μg/mL did not significantly alter the growth of these two cell lines relative to the vehicle (treated with DMSO alone). To eliminate the influence of cell death on observed parameters of cell migration and invasion, this concentration range was used in all subsequent experiments.

### 2.3. Inhibitory Effect of SOE on Migration of Endometrial Cancer Cells

**Figure 3:**
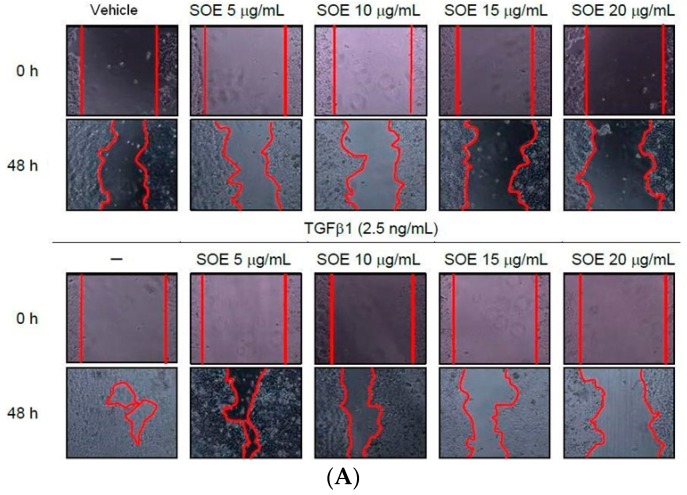

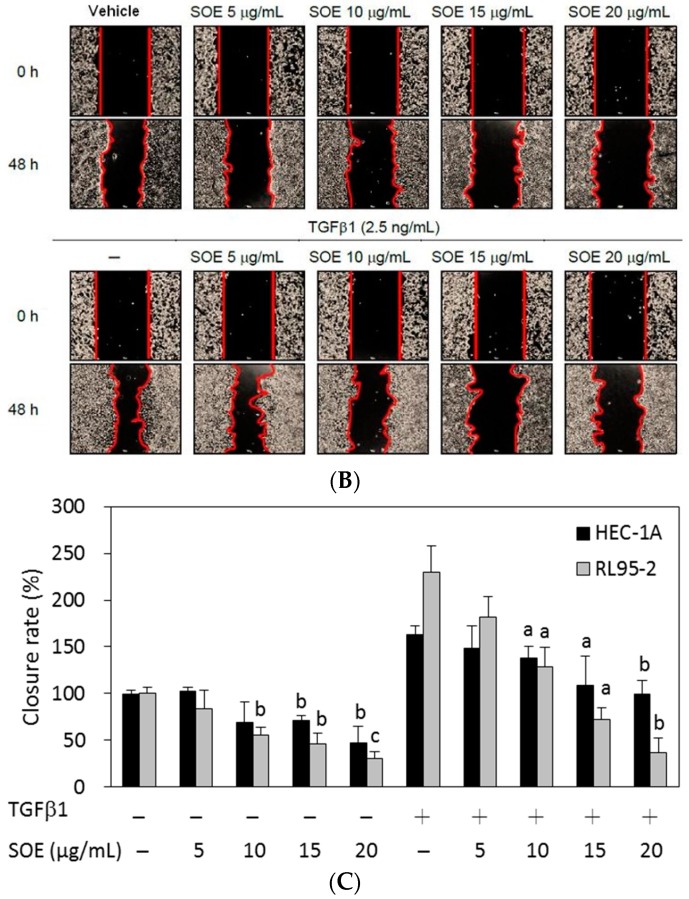
Effects of SOE dose on the motility of endometrial cancer cells. (**A**) HEC-1A cells; (**B**) RL95-2 cells; and (**C**) closure rates of both cell lines. Cancer cells were cultivated with a Culture Insert-2 Well (ibidi GmbH, Munich, Germany) for 24 h. After the culture insert was removed, DMEM with specified SOE concentrations was added, with or without TGFβ1 (2.5 ng/mL) stimulation, and the cells were then incubated. Photomicrographs of culture plates were directly obtained using a phase-contrast microscope (magnification 20×). Closure rate is defined as 100% without TGFβ1 induction and without SOE treatment. Three independent experiments were conducted; results are expressed as mean ± SD. A significant difference from the respective vehicle, according to Student’s *t*-test, was indicated as “a” for *p* < 0.05; “b” for *p* < 0.01, and “c” for *p* < 0.001.

The inhibitory effects of SOE on the motility of HEC-1A and RL95-2 cells were firstly studied using the wound healing assay. When treated with TGFβ1 in the absence of SOE (0 μg/mL), cells of both lines moved rapidly into the gap (Figure 3). Exposing the cells to a high concentration of SOE (15 and 20 μg/mL), with or without TGFβ1 induction, reduces their ability to migrate. These results indicate that SOE inhibited the migration of both cell lines in a dose-dependent manner, even under the induction with TGFβ1.

**Figure 4:**
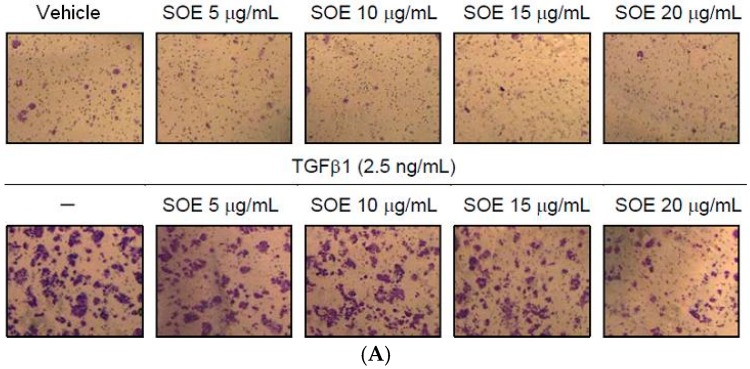

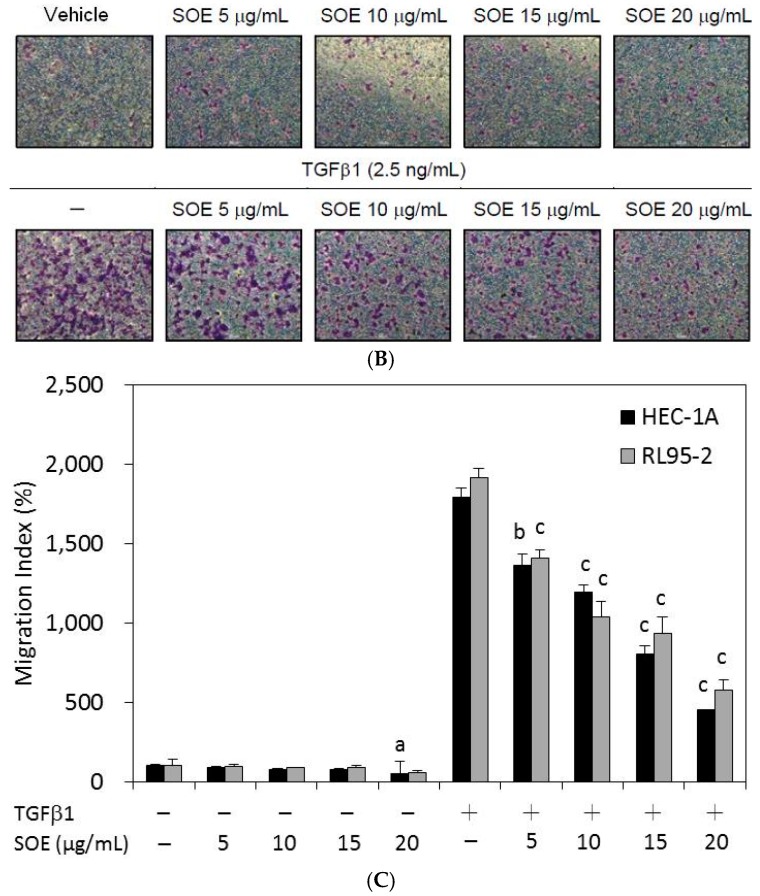
Effects of SOE dose on migration of endometrial cancer cells. (**A**) HEC-1A cells; (**B**) RL95-2 cells; and (**C**) the migration index of both cell lines. The migration assay was conducted using the Boyden chamber. HEC-1A and RL95-2 cells were treated with various doses of SOE for 48 h. Cells that penetrated the polycarbonate membrane to lower surface of the filter were stained with 0.5% crystal violet. The cell numbers were visualized under an inverted microscope with a magnification of 100× and were counted per field. The migration index is defined as 100% without TGFβ1 induction and without SOE treatment. Three independent experiments were performed; results are expressed as mean ± SD. A significant difference from the respective vehicle, according to Student’s *t*-test, was indicated as “a” for *p* < 0.05, “b” for *p* < 0.01, and “c” for *p* < 0.001.

A cell migration assay that used the Boyden chamber with a polycarbonate membrane was utilized to study further the effect of SOE on the motility of HEC-1A and RL95-2 cells. Cells were plated in the upper chamber, and the cells that moved to the underside of the coated polycarbonate membrane were counted under an optical microscope. SOE treatment significantly reduced the number of cells that migrated to the lower chamber (Figure 4). SOE retarded the migration of HEC-1A and RL95-2 cells in a dose-dependent manner relative to that of untreated controls. For the cells induced by TGFβ1, motility decreased to 25.2% (HEC-1A cells) and 30.1% (RL95-2 cells) after treatment with 20 μg/mL SOE for 48 h (*p* < 0.001).

### 2.4. Inhibitory Effect of SOE on Invasion of Endometrial Cancer Cells

**Figure 5:**
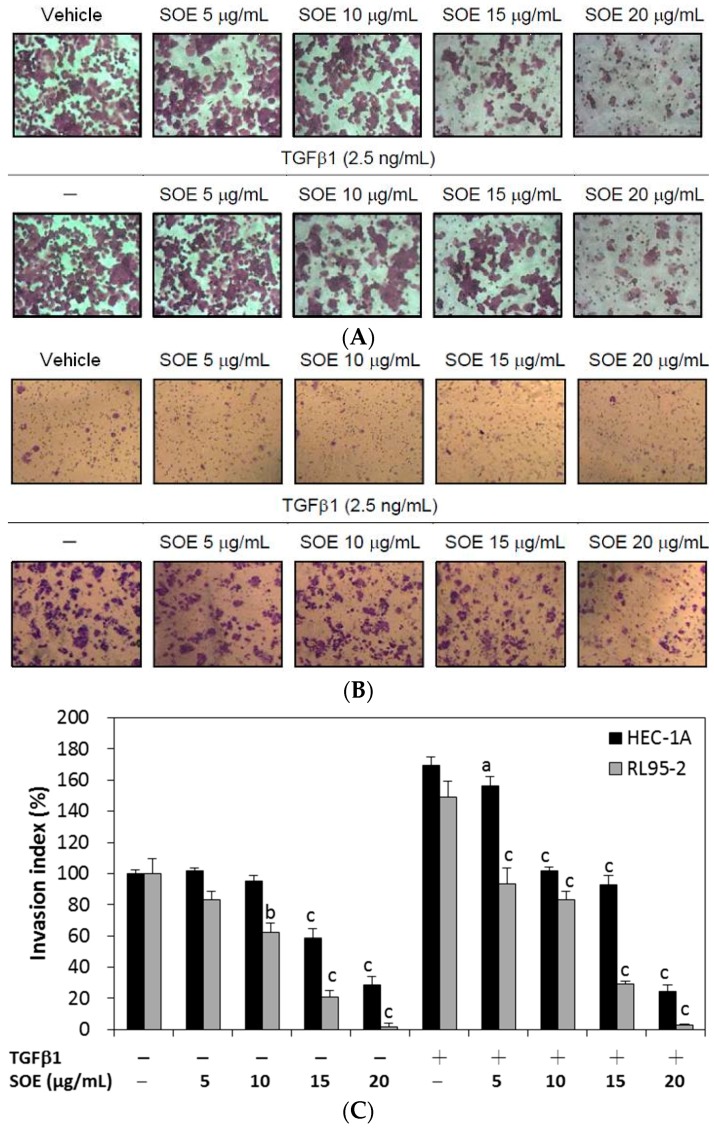
Effects of SOE dose on invasion of endometrial cancer cells. (**A**) HEC-1A cells; (**B**) RL95-2 cells; and (**C**) the invasion index of both cell lines. The invasion assay was conducted using the Boyden chamber. HEC-1A and RL95-2 cells were treated with various doses of SOE for 48 h. Cells that penetrated the Matrigel membrane to lower surface of the filter were stained with 0.5% crystal violet. The cell numbers were visualized under an inverted microscope with a magnification of 100× and were counted per field. The invasion index is defined as 100% without TGFβ1 induction and without SOE treatment. Three independent experiments were performed; results are expressed as mean ± SD. A significant difference from the respective vehicle, according to Student’s *t*-test, was indicated as “a” for *p* < 0.05; “b” for *p* < 0.01; and “c” for *p* < 0.001.

The progress of tumor invasion includes the degradation of basement membranes, the proteolysis of extracellular matrix (ECM), pseudopodial extension, and cell migration. The basement membrane, which is the first barrier of ECM to invasion by cancer, is composed of matrix macromolecules, such as type IV collagen, heparan sulfate proteoglycans, and laminin [27]. Thus, a Boyden chamber with a polycarbonate membrane that is coated with a collagen layer was used to investigate the inhibitory effect of SOE on invasion of endometrial cancer cells. SOE treatment of both cell lines, with and without TGFβ1 induction, significantly reduced the number of cells from the upper chamber that invaded the lower chamber (Figure 5). Quantification of cells in the lower chamber demonstrates that SOE treatment significantly inhibited the invasion of both cell lines in a dose-dependent manner. For the cells induced by TGFβ1, cell invasion was reduced to 14.4% (HEC-1A cells) and 1.7% (RL95-2 cells) after treatment with 20 μg/mL SOE for 48 h (*p* < 0.001).

### 2.5. Effects of SOE on Related Proteins Expression in RL95-2 Cells

The progress of tumor metastasis is regulated by complex mechanisms. The degradation of the ECM by increasing the expression and activation of extracellular proteases, such as metalloproteinases (MMPs), urokinase-type plasminogen activator (u-PA), or serine proteinase, has been regarded as important in cancer invasion and metastatic processes [28]. MMPs are the most essential proteases in the proteolysis of ECM proteins, such as collagen, fibronectin, proteoglycan, laminin, and elastin [29]. MMPs also play important roles in differentiation, proliferation, angiogenesis, and the destruction of inflammatory tissue [30,31]. Among the MMPs, MMP-2 (gelatinase A), and MMP-9 (gelatinase B) are the most essential in degrading of type IV collagen, which is the main constituent of the basement membrane [30]. Numerous papers have reported that the expressions of MMPs and u-PA correlate closely with the migration, invasion, and angiogenesis of various tumors [28,29,30,32].

Mitogen-activated protein kinases (MAPKs) are known to mediate and regulate cell growth, differentiation, apoptosis, and metastasis. The MAPK members, ERK1/2, p38 MAPK, and JNK/SAPK, have a critical role in regulating the expression of MMPs and u-PA [28,29,31,32]. Furthermore, the PI3K/Akt signaling pathway also plays a vital role in the regulation of MMPs and u-PA expression, and this pathway is associated with cell survival and metastasis of the development and progression of various tumors [29,32,33,34,35]. Recent studies have demonstrated that MAPK and PI3K/Akt signaling pathways are involved in the migration and metastasis of endometrial cancer cells [36,37].

**Figure 6:**
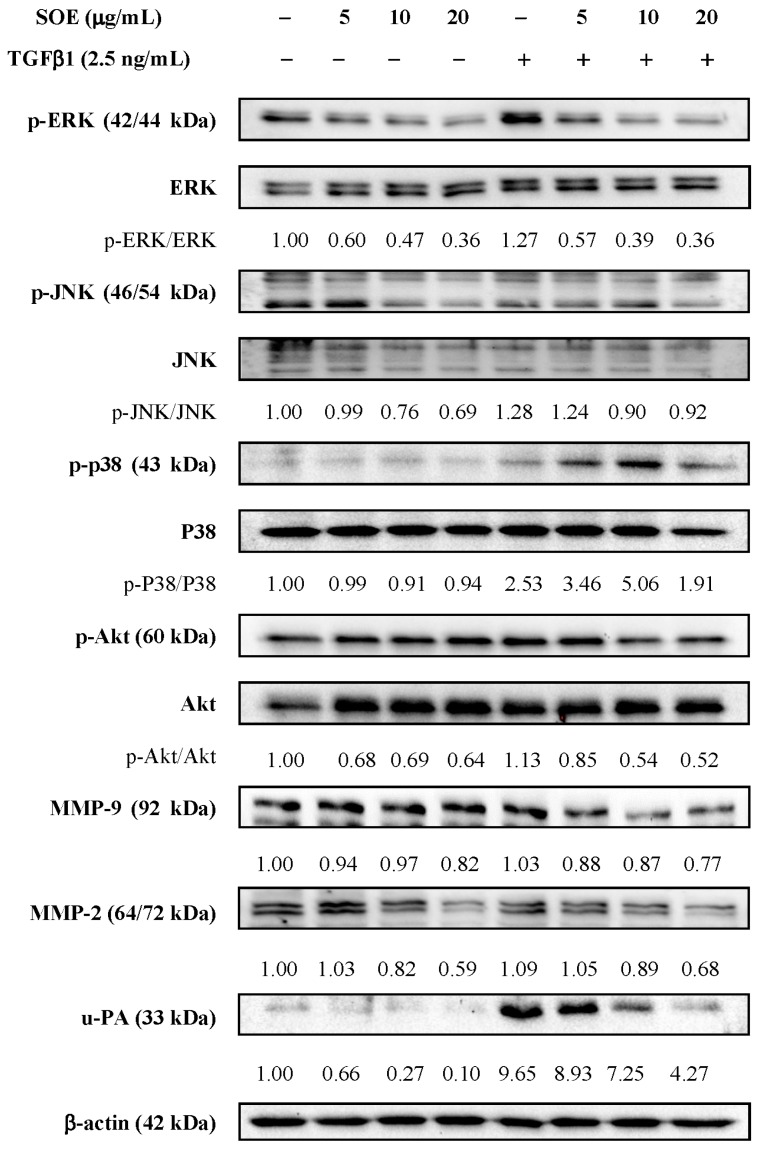
Western blot analysis of migration and invasion-related proteins in RL95-2 cells treated with different concentrations of SOE for 24 h. Whole-cell lysates were subjected to Western blot assays and β-actin was used as an internal control. The relative density of the proteins was determined by densitometric analysis. The values indicate the density proportion of each protein compared to that without TGFβ1 induction and without SOE treatment.

The expressed protein levels of p-ERK, p-JNK, p-p38, p-Akt, MMP-9, MMP-2, and u-PA following SOE treatment were evaluated by Western blotting. Regardless of whether the RL95-2 cells were stimulated by TGFβ1, Western blot analysis demonstrates that SOE treatment significantly reduced the levels of proteins p-ERK (42/44 kDa), p-JNK (46/54 kDa), p-Akt (60 kDa), MMP-9 (92 kDa), MMP-2 (64/72 kDa), and u-PA (33 kDa) in a dose-dependent manner (Figure 6). The SOE concentration range (5 to 20 μg/mL) that inhibited the expression of MAPKs, p-Akt, and MMPs coincided with the kinetics of cell migration and invasion.

## 3. Materials and Methods

### 3.1. Materials

The *S. orientalis* L. plant materials were purchased from Yuanshan Company (Kaohsiung City, Taiwan). Its nucleotide sequence was determined and deposited in the GenBank database with accession number JN987228 [38]. Dulbecco’s Modified Eagle Medium (DMEM) and fetal bovine serum (FBS) were from Gibco (Grand Island, NY, USA). TGFβ1 was purchased from R and D Systems, Inc. (Minneapolis, MN, USA). Antibodies against MMP-2 and MMP-9 were from Cell Signaling Technology (Beverly, MA, USA). Anti-actin antibody was purchased from BD Biosciences (San Jose, CA, USA). 3-(4,5-Dimethylthiazol-2-yl)-2,5-diphenyltetrazolium bromide (MTT) assay kit was from Sigma-Aldrich Chemicals (St. Louis, MO, USA).

### 3.2. Preparation of S. orientalis Ethanol Extract

The preparation of SOE was conducted as described previously [25]. In brief, dried powder (9.3 kg) of the aerial part of *S. orientalis* L. was extracted with 47 L of 95% ethanol for one day and this process was repeated three times. The extracted solutions were collected and filtered, and the solvent was removed using a rotary evaporator. The residue was then dried in a freeze-dryer. Total dry mass of this extract was 489 g. The chemical composition of the SOE has been reported elsewhere [26].

### 3.3. Cancer Cell Lines and Culture

Human endometrial cancer cell lines RL95-2 and HEC-1A were purchased from the American Type Cell Collection (ATCC, Manassas, VA, USA). Cells of these two cell lines were grown in DMEM that was supplemented with 10% (*v*/*v*) FBS, 1% penicillin/streptomycin and 0.02% sodium bicarbonate. These cancer cells were cultivated at 37 °C with 5% CO_2_ and 95% air, and in 100% relative humidity. The SOE was dissolved in dimethyl sulfoxide (DMSO) and the final DMSO concentration in the medium was less than 0.1%.

### 3.4. Determination of Cytotoxicity for Cancer Cells

Cancer cells were cultured in 96-well plates at 2 × 10^4^ cells/well and treated with the indicated concentration of SOE. After cultivation for the given period, the medium solution was removed. A 100 µL volume of the culture medium that contained 0.5 mg/mL of the MTT assay kit was added to each well. The cells were further cultured for 2 h, and the medium solution was removed. An aliquot of 100 µL DMSO was added and the plate was shaken until the crystals dissolved. The cytotoxicity against cancer cells was measured at 570 nm using an ELISA reader (Model 550, Bio-Rad Laboratories, Hercules, CA, USA).

### 3.5. Cell Migration by Wound Healing Assay

Cancer cells were seeded at 1 × 10^6^ cells/mL in 200 μL DMEM with a Culture Insert-2 Well (ibidi GmbH, Munich, Germany) and were cultivated for 24 h. After the culture insert was removed, DMEM with specified SOE concentration was added, with or without TGFβ1 (2.5 ng/mL) stimulation, and the cells were then incubated. Images of unfilled areas under each condition were obtained using an inverted phase-contrast microscope (Nikon Eclipse TS100, Chiyoda-ku, Tokyo, Japan) at a magnification of 20×. All wound healing assays were conducted in triplicate. The closure rate was calculated using the following formula:

Closure rate (%) = (Original width − Width after migration)/Original width × 100%(1)

### 3.6. Cell Migration and Invasion Assays by Boyden Chamber

Cell migration and invasion were assessed using the CytoSelect™ 24-well cell migration assay (Cell Biolabs, San Diego, CA, USA) and BioCoat™ Matrigel™ Invasion Chamber (BD Biosciences), respectively. To determine the effect of SOE on cell migration, 1 × 10^6^ cells/mL were seeded in serum-free DMEM on a polycarbonate membrane insert (8 μm pore size). The cells were then treated with various concentrations of SOE, with or without the stimulation by TGFβ1 (2.5 ng/mL). The bottom chamber was filled with DMEM that contained 10% FBS as a chemo-attractant. The chamber was incubated for 48 h at 37 °C. Next, non-migrating cells in the upper chamber were wiped away using a cotton-tipped swab, and invading cells were fixed with methanol, stained with 0.2% crystal violet, and photographed under a phase-contrast microscope. The same procedure was also carried out in experiments on cell invasion, except that the polycarbonate membrane was coated with a uniform layer of collagen. The migration index and the invasion index were estimated using the following equation:

Migration index or invasion index = Nu/Nc × 100%(2)

Nu: number of cells that moved to the underside of the membrane following SOE treatment; Nc: number of cells that moved to the underside of the membrane without TGFβ1 induction and SOE treatment.

### 3.7. Western Blot Analysis

To analyze the related proteins, 1 × 10^6^ cells were seeded into 6-cm culture dishes with or without SOE treatment, and with or without TGFβ1 stimulation. Following 24 h of incubation, the medium was removed and cells were washed several times using phosphate-buffered saline (PBS, 0.01 M, pH 7.2). Whole-cell lysates were prepared as described previously [26]. The harvested protein concentration was measured using a protein assay kit (Bio-Rad). Samples with equal amounts of denatured proteins were resolved on 10% sodium dodecyl sulfate polyacrylamide gel electrophoresis (SDS-PAGE). Proteins were transferred onto a nitrocellulose membrane (Immunobilin P; Millipore, Billerica, MA, USA), and were blocked for 1 h using 10% skimmed milk. The membranes were incubated overnight at 4 °C with the indicated primary antibodies. On the next day, the primary antibodies were washed away and secondary antibodies were added for incubation for 1 h at room temperature. The protein levels were detected using Enhanced Chemiluminescence (ECL) Plus Western blotting detection reagents (Amersham Bioscience, Uppsala, Sweden). Densitometric analyses were conducted using the Quantity One^®^ software (Bio-Rad).

### 3.8. Statistical Analysis

All experiments were carried out for three to five independent replicates. The experimental data were analyzed by using Microsoft Excel software (Microsoft Software Inc., Redmond, WA, USA). The data are expressed in terms of mean and standard deviation, and the statistical differences were analyzed by Student’s *t*-test.

## 4. Conclusions

In this study, SOE treatment effectively inhibited the migration and invasion of RL95-2 and HEC-1A endometrial cancer cells in a dose-dependent manner, with or without stimulation by TGFβ1, as determined by wound healing analysis and the Boyden chamber assay. SOE also reversed the TGFβ1-induced epithelial-mesenchymal transition. Western blot analysis demonstrated that SOE inhibited the phosphorylation of ERK1/2, JNK1/2, and Akt, as well as the expression of MMP-9, MMP-2, and u-PA in RL95-2 cells, in a dose-dependent manner. Since SOE also exhibits strong anti-inflammation and anti-proliferation activities, it may be a favorable complementary agent for treating patients with endometrial cancer.
